# Application of Autochthonous* Lactobacillus* Strains as Biopreservatives to Control Fungal Spoilage in Caciotta Cheese

**DOI:** 10.1155/2018/3915615

**Published:** 2018-07-16

**Authors:** Sofia Cosentino, Silvia Viale, Maura Deplano, Maria Elisabetta Fadda, Maria Barbara Pisano

**Affiliations:** Department of Medical Sciences and Public Health, University of Cagliari, Monserrato 09042, Italy

## Abstract

Fungal spoilage is one of the main causes of economic losses worldwide in the food industry. In the last years, consumer's demands for preservative-free processed foods have increased as a result of growing awareness about the health hazards associated with chemicals. Lactic acid bacteria have been extensively studied for their antibacterial and antifungal potential in order to be used as biopreservatives. The first objective of this study was to investigate* in vitro* the antifungal activity of autochthonous* Lactobacillus* strains against moulds commonly associated with cheese spoilage. Then, the* Lactobacillus* strains with the highest inhibitory effect and broadest spectrum were tested in single or mixed cultures against* Penicillium chrysogenum* ATCC 9179 and* Aspergillus flavus* ATCC 46283 on miniature Caciotta cheese produced at laboratory scale to evaluate* in situ* their ability to prevent mould growth and to determine their impact on cheese organoleptic properties and starter culture activity. The growth of the starter lactococcal population exhibited similar trend and values during ripening, suggesting that the addition of lactobacilli did not influence its growth and survival. Inhibition of* P. chrysogenum* inoculated in the milk was determined in cheeses produced with single or mixed* Lactobacillus* adjuncts as compared to cheeses without adjunct. The mixed adjunct cultures resulted in more effective, significantly reducing mould counts of more than 2 log units at the end of ripening. The application of the adjunct cultures resulted in a delay in mycelial growth of* P. chrysogenum* and* A. flavus* inoculated on the cheese surface as well. Finally, we found no significant differences among samples for the sensory parameters evaluated that received similar ratings. Our results indicate that the selected* Lactobacillus* strains may have a potential effect in controlling mould contamination on cheeses. Further studies are currently being carried out to identify the molecules responsible for the antifungal activity.

## 1. Introduction

Moulds and yeasts represent the main spoilage organisms of various foodstuff such as fermented dairy products (cheese and yogurt), bread, and stored crops [1]. Due to their low pH and water activity, nutritional profile, and storage at refrigeration temperatures, cheeses are very susceptible to the growth of filamentous fungi, in particular species of* Alternaria*,* Penicillium*,* Aspergillus*,* Cladosporium*,* Fusarium*,* Mucor*, and* Geotrichum* [2, 3]. The resulting products defects include visible surface growth of moulds that can cause discoloration, off-flavours, and alterations in the cheese rind and texture leading to significant economic losses. Some of these spoilage moulds may also produce mycotoxins, which are known to be potentially dangerous for public health [4]. Therefore, fungal spoilage represents a major cause of concern for the dairy industry.

Spoilage of cheese by moulds can be reduced using antifungal agents such as benzoic acid, sodium benzoate, potassium sorbate, and natamycin [5], but an increasing number of fungal species are becoming resistant to antimicrobials and preservatives [6, 7]. In addition, consumer demands for high-quality, preservative-free, and safe foods with an extended shelf-life raise the need to look for new preservation methods to control the growth of undesirable contaminating fungi.

Lactic acid bacteria (LAB) occur naturally in many foods and have a long history of safe use in the manufacture of dairy and other fermented products, demonstrated by the attribution of QPS (Qualified Presumption of Safety, in EU) and GRAS (Generally Recognized as Safe, in US) status [8, 9]. In addition, because of the increasing evidence on their positive health effects and their ability to produce a variety of antimicrobial compounds they could be considered as good candidates for cheese biopreservation in alternative to chemicals.

The antifungal activity of LAB has been attributed to the synergistic action of several compounds, e.g., organic acids (acetic, lactic, propionic, and phenyllactic acids), hydrogen peroxide, cyclic dipeptides, proteinaceous compounds, and fatty acids [10, 11], and it is known that the ability to synthetize these compounds is a strain-linked feature. Among LAB, several strains of the genus* Lactobacillus,* commonly found in cheese as the predominant nonstarter LAB, have been shown to possess specific antifungal activities and some are included in commercial protective cultures available in the market [12]. The limited number of marketed protective cultures in fermented dairy products may be related to the difficulty in finding strains possessing several important properties in addition to antimicrobial activity, such as the ability to growth in the desired food under manufacturing condition without producing any detrimental effect on the growth and functionality of the starter culture and without impairing the sensory attributes of the product.

Although the number of published studies on antifungal activity of LAB is increasing [11], the majority generally deal with the* in vitro* inhibitory properties of strains, while limited work has been carried out so far investigating the efficiency of LAB in controlling fungal growth in cheese manufacture and even fewer have evaluated the sensory characteristics of resultant cheeses or their possible impact on the activity of the starter cultures.

The objective of this study was to investigate the antifungal activity of autochthonous* Lactobacillus* strains against moulds commonly associated with cheese spoilage. The strains with the best* in vitro* activity were then used as adjunct, in single or mixed culture, in the manufacturing of Caciotta cheese at laboratory scale, in order to evaluate their ability to prevent mould growth and to determine their impact on cheese organoleptic properties using sensory analyses.

## 2. Materials and Methods

### 2.1. Microorganisms and Cultivation Conditions

A total of 22* Lactobacillus* strains (9* L. plantarum*, 6* L. paracasei*, 4* L. brevis*, and 3* L. sakei*) belonging to the Culture Collection of the Department of Medical Sciences and Public Health (CC-DSMSP, University of Cagliari) were selected for their wide* in vitro* antimicrobial properties as shown in previous studies [15, 16]. They were isolated from raw milk, artisanal ewes' cheeses, and sausages produced in Sardinia (Table 1) and were identified on the basis of phenotypic tests and genetic analysis based on polymerase chain reaction amplification using species-specific primers derived from 16S rRNA sequences (16S rDNA sequencing).

The moulds indicator strains used in the antifungal assays were from the American Type Culture Collection (ATCC) or the CC-DSMSP and were represented by 7 species commonly occurring in the environment, in cheese spoilage, or able to produce mycotoxins.

A commercial mesophilic homofermentative starter culture, including* Lactococcus lactis* subsp.* lactis* Lyoto MO540 and* Lactococcus lactis* subsp.* lactis* Lyoto MO536, provided by a dairy farm (Argiolas Formaggi, Dolianova, Cagliari, Italy) was used for cheesemaking trials.

*Lactobacillus* strains were maintained at −20°C in De Man Rogosa Sharpe (MRS) broth (Microbiol, Cagliari, Italy) with 15% (v/v) glycerol and routinely grown on MRS agar plates under microaerophilic conditions for 48 h at 30°C.

Fungi were stored in Potato Dextrose Broth (Microbiol, Cagliari Italy) with 20% glycerol at −20°C and subsequently grown on Potato Dextrose Agar plates (PDA, Microbiol) at 25°C for 7 days until sporulation occurred. Spores suspensions were prepared in physiological sterile solution with 0,5% Tween 80 (Microbiol).

### 2.2. In Vitro Antifungal Activity of LAB

Antifungal activity of* Lactobacillus* strains against* Alternaria alternata* (DSPMCM 109),* Cladosporium herbarum* (DSPMCM 110),* Paecilomyces variotii* (DSPMCM 18), and* Penicillium chrysogenum* ATCC 9179 indicator strains was tested* in vitro* using the agar plate method described by Guo et al. [13] with some modifications. Briefly, 100 *µ*l of fungal spore-mycelia suspension (approx. 10^4^ cfu/ml) was spread onto the surface of petri dishes containing 20 ml of modified MRS agar (mMRS: pH 6.0, sodium acetate and potassium dihydrogenphosphate omitted). After 30 min, bacteria were inoculated as two parallel lines of 3 cm length, keeping a distance between the lines of approximately 2 cm. Plates were incubated under microaerophilic conditions at 30°C for 48 h followed by an additional incubation under aerobic conditions at 25°C for 7 days to promote fungal growth.

In order to allow selected* Lactobacillus* strains to produce sufficient amount of inhibitory substances, the dual-culture overlay assay reported by Magnusson et al. [14] with some modifications was used to analyze the inhibitory activity against the fungal strains* Aspergillus flavus* ATCC 46283,* Fusarium oxysporum* (DSPMCM 31), and* Mucor recurvus* (DSPMCM 2), whose growth was much faster than that of lactobacilli. Briefly, bacteria were inoculated in 2 cm lines on MRS agar plates and allowed to grow at 30°C for 48 h in microaerophilic conditions. The plates were then overlaid with 7 ml of Sabouraud soft agar (Microbiol, 1% agar), containing 10^4^ spores per ml, and incubated in aerobiosis at 30°C for five to seven days.

For all assays, the antifungal activity of each LAB was ascertained by measuring the size of the halo surrounding the bacterial streaks, according to the following semiquantitative scale:

  +++: inhibition zone around* Lactobacillus* culture ≥ 8 mm
  ++: inhibition zone around* Lactobacillus* culture 5-7 mm
  +: inhibition zone around* Lactobacillus* culture 3-4 mm
  -: inhibition zone around* Lactobacillus* culture < 3 mm.

All the experiments were performed in duplicate.

### 2.3. Miniature Caciotta Cheese Manufacture and In Situ Antifungal Activity of LAB

Four* Lactobacillus* strains with the highest* in vitro* inhibitory effect were used as adjunct in the manufacturing of Caciotta cheese at laboratory scale, in order to evaluate their ability to inhibit* Penicillium chrysogenum* ATCC 9179 and* Aspergillus flavus* ATCC 46283 strains.

**Figure 1:**
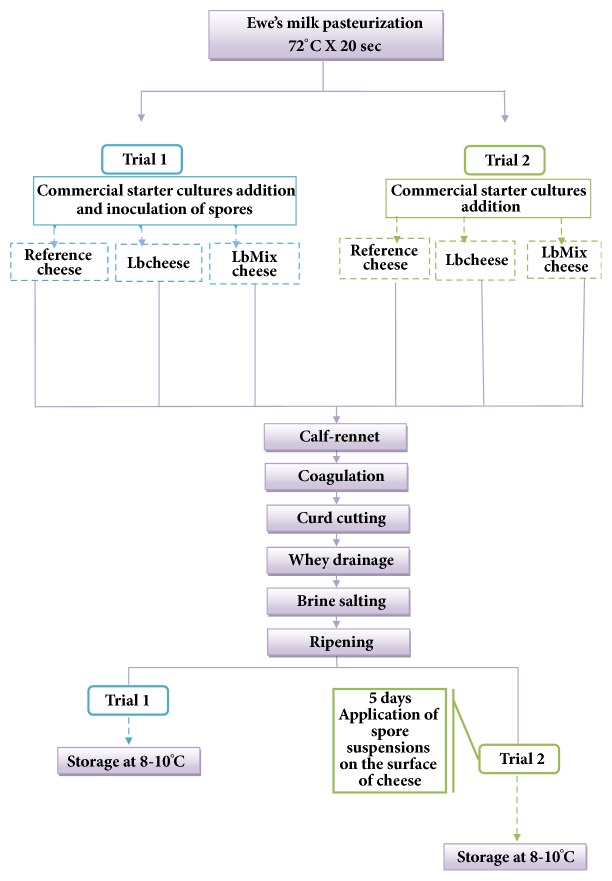
Protocol used in the manufacturing of miniature Caciotta cheeses.

Miniature Caciotta cheese was manufactured under aseptic conditions following the protocol reported in Figure 1.

Two different cheesemaking trials were performed. In each trial, three cheese batches were simultaneously produced with the same pasteurized ewes' milk obtained from a local dairy farm (Argiolas Formaggi): one batch containing only the commercial starter culture (reference cheese); a second batch containing the commercial starter and the* L. plantarum* C1col15 strain (Lb cheese); a third batch containing the commercial starter with the addition of a multi-*Lactobacillus* adjunct (LbMix cheese), containing the following strains:* L. plantarum* 4/16898,* L. plantarum* 1/14537, and* L. brevis* DSM 32516. Fresh lactobacilli cultures were prepared in autoclaved reconstituted skimmed milk after two consecutive transfers in MRS broth (1% inoculum) incubated at 30°C in aerobic conditions for 18 h. Ten cheeses for each batch were produced in each trial.

The mean composition of raw ewe's milk used for cheesemaking was 6.43% fat, 5.58% protein, and 4% lactose, and the pH measured at 6.7.

In the first trial (T1), the commercial starter culture was inoculated (1% v/v) at a level of 7 log CFU/mL to 45 L of pasteurized milk, followed by the inoculation of a* P. chrysogenum* ATCC 9179 spore suspension (10^2^ cfu/ml). After that, the milk was divided into three batches of approximately 15 L each: one was inoculated with the single culture (Lb cheese), one was inoculated with the mixed adjunct (LbMix cheese), and the last was considered as the control (reference cheese, without any added* Lactobacillus* culture). After 30 min of resting time, liquid rennet was added to the milk at level of 0.1 ml/L and coagulation took place at 37°C within 15 min. The coagulum was cut manually using a sterile steel knife and the curd was left to rest for 10 minutes. Then, the curd pieces were hand-pressed into moulds for whey drainage (25°C). After brine salting for 20 min (NaCl 30%), the cheeses were ripened at 8-10°C for 1 month. The weight of the cheeses was about 190 g.

The second trial (T2) was carried out with the protocol described above but instead of spore inoculation in milk, the moulds* P. chrysogenum* ATCC 9179 and* A. flavus ATCC 46283* (10 *µ*l of a suspension containing 10^4^ cfu/ml fungal spores) were applied on the surface of 5-day cheeses. Of the ten cheeses manufactured in the three batches, eight were inoculated (four with* Penicillium *and four with* Aspergillus*) and two were left uninoculated to serve as control, in order to assess if the antifungal strains were able to inhibit airborne mould growth and to be used for sensory analysis.

During ripening time, the cheeses were checked periodically in order to monitor fungal growth and were photographed with a digital camera.

Samples were taken for microbiological analyses after 5, 15, and 30 days of ripening in T1 and after 15 and 30 days in T2.

### 2.4. Microbiological Analyses

Microbiological characteristics were analysed to evaluate the effectiveness of fungal biopreservation on cheese during ripening time. Duplicate ten grams aliquots of cheese were transferred to a sterile tube containing 90 ml of 2% (w/v) sodium-citrate sterile solution. Cheese samples were homogenized in a Stomacher Lab Blender (Pool Bioanalysis Italiana, Milan, Italy) for two minutes at normal speed. Decimal dilutions were prepared in sterile solution of 0.1% (w/v) peptone and spread onto the surface of the different agar media. Lactococci were enumerated in M17 agar (Microbiol) incubated at 30°C for 48 h and lactobacilli in MRS agar acidified at pH 5.4 with glacial acetic acid incubated at 30°C in microaerophilic conditions for 48 h. Yeasts and moulds were counted in PDA plates containing 0.1 g/L chloramphenicol incubated at 25°C for 5 to 20 days.

The pH of cheeses was measured with a HI8520 pH meter (Pool Bioanalysis Italiana).

### 2.5. Sensory Analysis

At 30 days of ripening, the cheese samples manufactured in the second trial and not surface inoculated with fungal spore suspension were subjected to sensory evaluation by 10 untrained panelists recruited among regular cheese consumers. The sensory evaluation was conducted with the aim of estimating the differences in the cheeses manufactured with adjuncts cultures compared with the reference cheese and detecting off-flavours and defects eventually caused by the adjuncts. The qualities judged were cheese shape, odour, flavour, and paste color and texture, scoring on a scale from 4 to 10 (4: very poor, 10: very good). Representative slices of 2 cm cheese samples were cut and placed in closed individual petri dishes for 2 h before evaluation. Each tester was served the three cheese samples coded with a three-digit code number and presented in random order.

### 2.6. Statistical Analyses

Microbial counts were calculated as number of colony forming units (cfu) per gram of sample and reported as log_10_ cfu/g or ml. The data obtained from microbiological and sensory analyses were evaluated by one-way analysis of variance (ANOVA) and Tukey's test using GraphPad Prism Statistics software package version 3.00 (GraphPad Prism Software Inc., San Diego, CA, USA), to determine the differences among the means. Statistical significance was inferred at P < 0.05.

## 3. Results and Discussion

### 3.1. In Vitro Antifungal Activity of LAB

The* Lactobacillus* strains investigated in this study were previously characterized in order to evaluate their potential for using as adjunct cultures in the manufacturing of cheese and were shown to possess wide antibacterial properties [15, 16].

These strains were first tested for their* in vitro* antifungal activity against seven mould species chosen because of their common occurrence in cheese spoilage and ability to produce mycotoxins [3, 4].

As shown in Table 1, all strains were active against at least two mould species, with the exception of* L. brevis* 9/FSNS11B that showed no activity whatsoever, and the majority displayed a broad antifungal spectrum. The antifungal ability was dependent on both fungal species and* Lactobacillus* strain:* P. chrysogenum* ATCC 9179 was the most sensitive indicator strain, being strongly inhibited (inhibition zone higher than 8 mm) by the majority of strains while* P. variotii* (DSPMCM 18) and* M. recurvus* (DSPMCM 2) were the least sensitive and the highest inhibition activity was observed for* L. plantarum*, followed by* L. sakei*,* L. brevis* and* L. paracasei*. Three* L. plantarum* (4/16868, 1/14537, and C1col15) and one* L. brevis* (DSM 32516) strains were strongly active against all moulds tested, the latter with a lower inhibition activity against only* C. herbarum* (DSPMCM 110). Varying degrees of inhibition were observed for the other* Lactobacillus* strains.

Although the* in vitro* antifungal activity of LAB strains has been evaluated in several studies, comparison of results is often difficult, due to different strains, conditions of assays, and methods used. Fernandez et al. [17] found lactobacilli strains with strong antifungal activity against* P. chrysogenum*,* M. racemosus*,* A. versicolor*, and* C. herbarum*. In large screening of 897 LAB strains isolated from herbs, fruits, and vegetables, Cheong et al. [18] came across 12* L. plantarum* strains able to inhibit* P. solitum*,* A. versicolor*, and* C. herbarum*. Two probiotic* Lactobacillus* strains (*L. rhamnosus* L60 and* L. fermentum* L23) grown in coculture with aflatoxigenic* A. flavi* completely inhibited the fungal growth and aflatoxin B1 production [19].

In agreement with our findings, the antifungal activity of* L. plantarum* has been reported by other authors [20–22] and* L. plantarum* strains have been investigated as mould controlling agents in different foods [23–25]. Beside* L. plantarum*, most of the active antifungal strains in fermented milk products according to the literature are related to the* L. casei* group [26], while fewer studies have dealt with the inhibitory activity of* L. sakei* and* L. brevis.* Voulgari et al. [27] found several* L. paracasei* strains of dairy origin active against* P. candidum*, in contrast with our results showing this species as the least effective against the moulds tested. In the study by Tropcheva et al. [28] four* L. brevis* isolates from the traditional Bulgarian dairy product “katak” were characterized as cultures with promising antifungal activity. Two strains of* L. sakei* were shown to possess high or moderate activity against the moulds* P. commune*,* A. fumigatus*,* A. nidulans*, and* F. sporotrichioides* by Magnusson et al. [14].

### 3.2. Antifungal Activity of LAB in Cheese

In the second part of the study, the four* Lactobacillus* strains with the highest inhibitory effect and the broadest spectrum, namely,* L. plantarum* 4/16868, 1/14537, C1col15, and* L. brevis* DSM 32516, were tested in single or mixed cultures against* P. chrysogenum* ATCC 9179 and* A. flavus* ATCC 46283 (aflatoxin B1 producer) on miniature Caciotta cheese produced under laboratory conditions, in order to evaluate* in situ* their potential as biopreservatives. In all trials, cheese without added* Lactobacillus* adjunct was used as control (reference cheese).

Tables 2 and 3 report the evaluation of pH and the results of microbiological analyses carried out during ripening on samples from trials T1 and T2, respectively. In all cheeses, regardless of trial protocol, the growth of the starter lactococcal population exhibited similar trend and values during ripening, suggesting that the addition of lactobacilli adjunct did not influence its growth and survival.

The pH of all cheeses generally decreased during ripening, reaching mean values between 5.01 and 4.72 in trial T1 (reference cheese and Lb cheese at 30 days) and between 5.82 and 4.74 in trial T2 (reference control cheese and LbMix cheese at 30 days). Cheeses produced with adjunct cultures showed significantly lower values with respect to reference cheeses, probably due to the combined acidifying activity of starter and lactobacilli. In fact, lactococcal starter cultures and lactobacilli are known to produce organic acids during cheese maturation that are presumably responsible for the pH decrease. Similar findings were reported by Ulpathakumbura et al. [29] in cheddar cheese.

As for lactobacilli trend,* Lactobacillus* adjuncts were added to the milk at 10^7^ cfu/ml and their level increased about 1 or 2 log unit at the end of the ripening period. The control cheeses without added adjuncts were free from lactobacilli (counts below the detection limit of our method), showing the efficacy of our laboratory work conditions used to avoid contamination. The colonies counted as presumptive lactobacilli were randomly picked and identified by biochemical and molecular means that confirmed their belonging to the L*actobacillus* species inoculated (data not shown).

**Figure 2:**
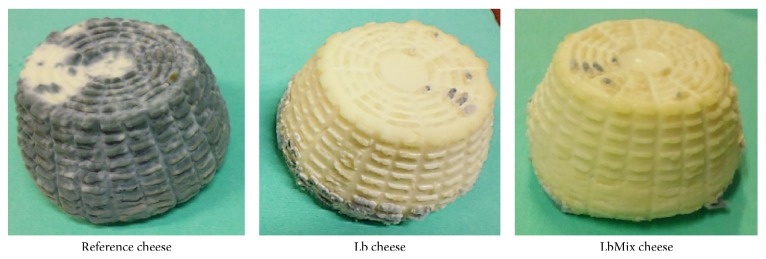
Photographs showing the antifungal effect of* Lactobacillus* adjuncts on cheeses produced with (Lb and LbMix) and without (Reference) antifungal cultures, in trial T1 (inoculation of* P. chrysogenum* ATCC 9179spores in milk) at 30 days of ripening at 8°C.

In trial T1 (Table 2 and Figure 2), a significant growth inhibition of* P. chrysogenum* inoculated in the milk was determined in Caciotta cheese produced with single or mixed* Lactobacillus* adjunct culture (Lb and LbMix cheeses, respectively) as compared to cheeses without adjunct. In all cheeses, the fungus reached maximum numbers at the end of ripening (30 days), and the mixed adjunct cultures resulted more effective, reducing mould counts of more than 2 log units after 15 and 30 days of ripening.

As demonstrated by other authors [30, 31], the antifungal activity of protective cultures depends on the initial numbers of competitive bacteria. In our study, the inoculation of lactobacilli at a concentration of 10^7^ cfu/ml into the milk at the same time as* P. chrysogenum* was able to retard fungal growth as compared to a control without adjunct culture, in agreement with the results obtained by Lacanin et al. [31] in a yogurt model. Even though, as stated above, comparison with the literature is difficult, our results are in agreement with those of Cheong et al. [18] that found several strains of* L. plantarum* able to prevent the visible growth of* P. commune* on cottage cheese between 14 and > 25 days longer than control (cheese without added antifungal LAB). The ability of* L. plantarum* strains to inhibit mould growth in different types of foods such as bread [32], fresh vegetables [33], and fruits [34] has been demonstrated. Fernandez et al. [17] were able to inhibit* P. chrysogenum* growth for at least 21 days at 6°C in cottage cheese using* L. rhamnosus* A238 alone or in combination with* Bifidobacterium animalis* subsp.* lactis* A026.

**Figure 3:**
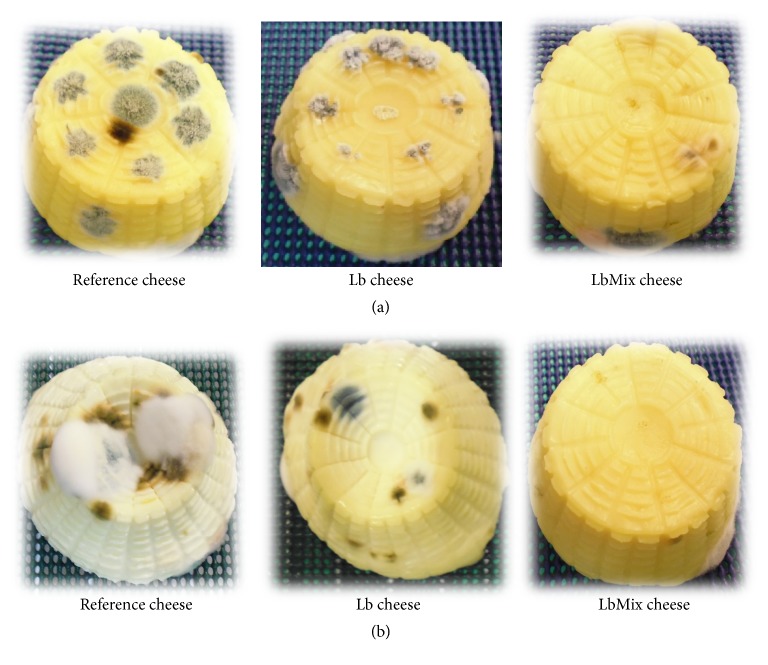
Photographs showing the antifungal effect of* Lactobacillus* adjuncts on cheeses produced with (Lb and LbMix) and without (reference) antifungal cultures, in trial T2 (cheese surface inoculation of spores) at 30 days of ripening at 8°C. (a) Inoculation of* P. chrysogenum* ATCC 9179; (b) inoculation of* A. flavus* ATCC 46283.

The results of trial T2 are shown in Table 3 and Figure 3. The application of the adjunct cultures (single or mixed) resulted in a delay in mycelial growth of* P. chrysogenum* and* A. flavus* inoculated on the cheese surface (10^4^ spores/ml), but overall the level of inhibition was lower with respect to trial T1. Again, the LbMix culture exhibited the strongest antifungal activity. The higher effectiveness of the mixed-strains culture as compared to the single one might be related to the combined synergy of multiple compounds, as already reported [35]. Finally, both adjuncts were able to prevent airborne mould growth in control cheese. After applying moulds suspensions on cheese surfaces, Lynch et al. [36] determined a 6-day delay of* Penicillium* growth using* L. amylovorus* as adjunct culture in cheddar cheese, while in the study by Sedaghat et al. [25] the application of* L. plantarum* strains as fresh cheese starter culture resulted in a significant delay in mycelial growth of* A. flavus* and* A. parasiticus* on cheese surface.

### 3.3. Sensory Properties

Taste and aroma are recognized as important features for determining cheese quality and identity. In our study, there was no significant difference among samples for all the parameters evaluated (p > 0.05) that received similar ratings. Cheese made with LbMix adjunct received the highest score for flavour (Table 4). Since the cheese flavour is known to be related to lipolysis and proteolysis by starter and NSLAB cultures, the cooperation between the starter and the mix of lactobacilli used in this cheese-batch could have increased flavour development, but this needs to be demonstrated in further experiments. Several adjunct cultures of* L. plantarum* species have been claimed to increase peptidolysis and improve sensory properties of cheese [37]. To our best knowledge, very few studies have investigated the sensory properties of cheeses manufactured with antifungal LAB adjuncts. In the work of Ulpathakumbura et al. [29], Cheddar cheese made with* L. rhamnosus* as adjunct culture received lower sensory ratings with respect to nisin-incorporated cheese samples, whereas a strain of* L. harbiniensis* at an inoculation rate of 5 × 10^6^ cfu/ml in milk had no detrimental effect on yogurt organoleptic properties [38].

## 4. Conclusions

In order to investigate their effectiveness as cheese preservatives to extend the shelf-life and prevent the fungal spoilage during storage at 8°C,* Lactobacillus* strains of food origin were first screened against a variety of moulds commonly associated with cheese contamination and spoilage to evaluate their antifungal activity spectrum; then the four most effective strains were tested against* Penicillium* and* Aspergillus* on miniature Caciotta cheese.

The addition of selected cultures was able to delay, to varying degree, the mycelial growth of both* P. chrysogenum* ATCC 9179 and* A. flavus* ATCC 46283 as well as that of environmental fungi on the cheese surface, although none was able to prevent totally, in any experimental conditions, the growth of targeted moulds.

The strongest antifungal effect was observed in the cheese produced with a multi-*Lactobacillus* strains adjunct (LbMix) when the mould* P. chrysogenum* ATCC 9179 was inoculated in the milk.

Our results indicate that the selected* Lactobacillus* strains may have a potential effect in controlling the mould contamination on cheeses without altering the sensory characteristics when used in coculture with the starter* L. lactis*. However, their bioprotective activity in cheese needs to be confirmed at industrial level. Further studies are currently being carried out to identify the molecules responsible for the antifungal activity of these strains.

## Figures and Tables

**Table 1:** *In vitro* inhibition of *Lactobacillus* strains isolated from Sardinian dairy products against the fungal indicator strains tested.

		Indicator strains						
Strains	Origin	*A. alternata* (DSPMCM 109)	*P. chrysogenum* ATCC 9179	*P. variotii* (DSPMCM 18)	*C. herbarum* (DSPMCM 110)	*M. recurvus* (DSPMCM 2)	*A. flavus* ^*∗*^ ATCC 46283	*F. oxysporum* (DSPMCM 31)
*L. plantarum *11/20966	Ewe's milk	-	+++	+	+	+	+	+
*L. plantarum *4A/20045	Ewe's milk	-	-	-	-	+	+	-
*L. plantarum *1B3M	Ewe's cheese	-	-	-	+	-	-	+
*L. plantarum *4/16868	Ewe's milk	+++	+++	+++	+++	+++	+++	+++
*L. plantarum *19/20711	Ewe's milk	-	+++	-	-	-	++	+++
*L. plantarum *1/14537	Ewe's milk	+++	+++	+++	+++	+++	+++	+++
*L. plantarum *3/15919	Ewe's milk	+	+++	-	+	-	+	-
*L. plantarum *C1col15	Ewe's cheese	+++	+++	+++	+++	+++	+++	+++
*L. plantarum *10B3M	Ewe's cheese	-	+	-	+	-	-	-
*L. paracasei *31LP27	Ewe's milk	+	+	+	+	+	+	+
*L. paracasei* 15/FS153M	Ewe's cheese	-	+	+	-	+	-	+
*L. paracasei *19/FS151M	Ewe's cheese	+	+++	+	-	-	-	+++
*L. paracasei *1A6M	Ewe's cheese	-	-	-	-	-	-	++
*L. paracasei *8/18710	Ewe's milk	-	-	-	+	-	+	-
*L. paracasei *28/10A	Ewe's cheese	+	+++	+	+	+	+	-
*L. brevis *DSM 32516	Ewe's cheese	+++	+++	++	+	+++	+++	+++
*L. brevis *3/FSNS11A	Ewe's cheese	+	+	+	+	+	+	-
*L. brevis *9/FSNS11B	Ewe's cheese	-	-	-	-	-	-	-
*L. brevis *S1	Sausage	+	+	-	-	-	+	-
*L. sakei *S5	Sausage	++	+++	+	++	++	++	++
*L. sakei *S3	Sausage	+++	+	++	+++	++	+	++
*L. sakei *S4	Sausage	+++	++	+	+++	+	++	+++

Inhibition tested according to Guo et al. [13] for *A. alternata, P. chrysogenum, P. variotii, *and* C. herbarum*.

Inhibition tested according to Magnusson et al. [14] for *M. recurves, A. flavus, *and* F. oxysporum*.

Inhibition was scored by measuring the size of the halo around the bacterial streaks according to the following semiquantitative scale: (+++) inhibition zone ≥ 8 mm; (++) inhibition zone 5-7 mm; (+) inhibition zone 3-4 mm; (-) inhibition zone < 3 mm.

^*∗*^Aflatoxin B1 producer.

**Table 2:** Evolution of pH and viable counts (log cfu/g) of moulds, lactococci, and lactobacilli in cheeses produced with (Lb and LBMix) and without (reference) antifungal cultures, in trial T1 (inoculation of *P. chrysogenum* spores in milk) during ripening at 8°C.

		Days of ripening		
	Cheese type	5	15	30
Moulds (PDA)	Reference	2.89 ± 0.03^a^	7.33 ± 0.08^a^	8.15 ± 0.05^a^
	Lb	2.39 ± 0.05^b^	5.71 ± 0.01^b^	6.06 ± 0.06^b^
	LbMix	<2	4.80 ± 0.03^c^	5.90 ± 0.01^c^

Presumptive lactococci (M17)	Reference	6.49 ± 0.04^a^	7.37 ± 0.04^a^	7.56 ± 0.04^a^
	Lb	6.48 ± 0.02^a^	7.36 ± 0.02^a^	7.57 ± 0.06^a^
	LbMix	6.50 ± 0.02^a^	7.45 ± 0.08^a^	7.58 ± 0.05^a^

Presumptive lactobacilli (MRS)	Reference	<2	<2	<2
	Lb	8.12 ± 0.03^a^	9.05 ± 0.03^a^	9.30 ± 0.02^a^
	LbMix	7.99 ± 0.01^b^	8.42 ± 0.16^b^	9.86 ± 0.08^b^

pH	Reference	5.05 ± 0.06^a^	5.02 ± 0.05^a^	5.01 ± 0.03^a^
	Lb	4.92 ± 0.03^b^	4.73 ± 0.05^b^	4.72 ± 0.03^b^
	LbMix	5.01 ± 0.01^a^	4.87 ± 0.06^c^	4.82 ± 0.06^b^

Values are the mean ± standard deviation of triplicate samples for each cheese type.

Different superscripts in the same column indicate significant differences (P < 0.05).

**Table 3:** Evolution of pH and viable counts (log cfu/g) of moulds, lactococci, and lactobacilli in cheeses produced with (Lb and LBMix) and without (Reference) antifungal cultures, in trial T2 (cheese-surface inoculation of spores) during ripening at 8°C.

	Cheese type	*P. chrysogenum * ATCC 9179		*A. flavus ATCC 46283* ^*∗∗*^		Control cheeses^*∗*^	
				Days of ripening			
		15	30	15	30	15	30
Moulds	Reference	8.80 ± 0.28^a^	9.30 ± 0.42^a^	8.60 ± 0.42^a^	9.80 ± 0.28^a^	3.54 ± 0.34^a^	4.50 ± 0.28^a^
	Lb	6.50 ± 0.71^b^	8.30 ± 0.42^a^	7.15 ± 0.11^a^	8.74 ± 0.37^a^	3.15 ± 0.11^a^	4.23 ± 0.11^a^
	LbMix	7.30 ± 0.42^b^	8.10 ± 0.14^a^	7.20 ± 0.57^a^	7.41 ± 0.28^b^	2.80 ± 0.28^a^	3.80 ± 0.28^a^

Presumptive lactococci	Reference	7.40 ± 0.01^a^	7.69 ± 0.01^a^	7.46 ± 0.04^a^	7.66 ± 0.02^a^	7.13 ± 0.32^a^	7.29 ± 0.40^a^
	Lb	7.37 ± 0.01^a^	7.60 ± 0.02^a^	7.37 ± 0.04^a^	7.61 ± 0.01^a^	7.00 ± 0.56^a^	7.30 ± 0.42^a^
	LbMix	7.39 ± 0.01^a^	7.61 ± 0.27^a^	7.38 ± 0.01^a^	7.60 ± 0.01^a^	7.21 ± 0.29^a^	7.30 ± 0.27^a^

Presumptive lactobacilli	Reference	<2	<2	<2	<2	<2	<2
	Lb	7.88 ± 0.16^a^	7.25 ± 0.07^a^	7.44 ± 0.21^a^	7.49 ± 0.15^a^	7.65 ± 0.49^a^	7.89 ± 0.58^a^
	LbMix	8.04 ± 0.13^a^	8.47 ± 0.01^b^	8.32 ± 0.03^b^	9.37 ± 0.04^b^	8.13 ± 0.25^a^	8.91 ± 0.13^a^

pH	Reference	5.68 ± 0.11^a^	5.58 ± 0.11^a^	5.53 ± 0.11^a^	5.25 ± 0.07^a^	5.91 ± 0.01^a^	5.82 ± 0.03^a^
	Lb	5.10 ± 0.14^b^	5.05 ± 0.07^b^	5.18 ± 0.04^b^	4.85 ± 0.07^b^	5.77 ± 0.03^b^	5.68 ± 0.11^a^
	LbMix	4.98 ± 0.02^b^	4.78 ± 0.02^b^	4.95 ± 0.07^b^	4.74 ± 0.08^b^	4.94 ± 0.03^c^	4.90 ± 0.01^b^

Values are the mean ± standard deviation of duplicate samples for each cheese type.

Different superscripts in the same column indicate significant differences (P < 0.05).

^*∗*^Uninoculated cheese samples: values are mean ± standard deviation of two aliquots of the same cheese sample.

^*∗∗*^Aflatoxin B1 producer.

**Table 4:** Sensory analysis of miniature Caciotta cheese made with (Lb and LBMix) and without (Reference) *Lactobacillus* adjunct cultures at 30 days of ripening at 8°C.

Sensory attributes					
Cheese type	Shape	Odour	Flavour	Paste colour	Paste texture
Reference	8.52 ± 0.34^a^	6.48 ± 0.31^a^	6.80 ± 0.75^a^	8.32 ± 0.45^a^	8.56 ± 0.53^a^
Lb	8.64 ± 0.49^a^	5.52 ± 0.96^a^	7.10 ± 1.37^a^	8.68 ± 0.50^a^	8.44 ± 0.51^a^
LbMix	8.44 ± 0.65^a^	6.49 ± 1.02^a^	7.60 ± 1.35^a^	8.40 ± 0.66^a^	8.44 ± 0.55^a^

Values are the mean ± standard deviation of ten evaluation for each cheese type.

Different superscripts in the same column indicate significant differences (P < 0.05).

## Data Availability

The mean ± standard deviation of microbial and sensory data used to support the findings of this study is included within the article. The raw data are available from the corresponding author upon request.
